# Multiscale modeling of neuronal dynamics in hippocampus CA1

**DOI:** 10.3389/fncom.2024.1432593

**Published:** 2024-08-06

**Authors:** Federico Tesler, Roberta Maria Lorenzi, Adam Ponzi, Claudia Casellato, Fulvia Palesi, Daniela Gandolfi, Claudia A. M. Gandini Wheeler Kingshott, Jonathan Mapelli, Egidio D'Angelo, Michele Migliore, Alain Destexhe

**Affiliations:** ^1^CNRS, Paris-Saclay Institute of Neuroscience (NeuroPSI), Paris-Saclay University, Gif-sur-Yvette, France; ^2^Department of Brain and Behavioural Sciences, University of Pavia, Pavia, Italy; ^3^Institute of Biophysics, National Research Council, Palermo, Italy; ^4^Digital Neuroscience Centre, IRCCS Mondino Foundation, Pavia, Italy; ^5^Department of Engineering “Enzo Ferrari”, University of Modena and Reggio Emilia, Modena, Italy; ^6^NMR Research Unit, Queen Square MS Centre, Department of Neuroinflammation, UCL Queen Square Institute of Neurology, Faculty of Brain Sciences, University College London, London, United Kingdom; ^7^Department of Biomedical, Metabolic and Neural Sciences, University of Modena and Reggio Emilia, Modena, Italy

**Keywords:** spiking neural network, hippocampus, mean-field, traveling waves, oscillations, multiscale

## Abstract

The development of biologically realistic models of brain microcircuits and regions constitutes currently a very relevant topic in computational neuroscience. One of the main challenges of such models is the passage between different scales, going from the microscale (cellular) to the meso (microcircuit) and macroscale (region or whole-brain level), while keeping at the same time a constraint on the demand of computational resources. In this paper we introduce a multiscale modeling framework for the hippocampal CA1, a region of the brain that plays a key role in functions such as learning, memory consolidation and navigation. Our modeling framework goes from the single cell level to the macroscale and makes use of a novel mean-field model of CA1, introduced in this paper, to bridge the gap between the micro and macro scales. We test and validate the model by analyzing the response of the system to the main brain rhythms observed in the hippocampus and comparing our results with the ones of the corresponding spiking network model of CA1. Then, we analyze the implementation of synaptic plasticity within our framework, a key aspect to study the role of hippocampus in learning and memory consolidation, and we demonstrate the capability of our framework to incorporate the variations at synaptic level. Finally, we present an example of the implementation of our model to study a stimulus propagation at the macro-scale level, and we show that the results of our framework can capture the dynamics obtained in the corresponding spiking network model of the whole CA1 area.

## 1 Introduction

The development of large-scale models and simulations of brain activity (going from thousands of neurons to full regions and whole-brain scale) has seen a great advance in the last few years, boosted by the increase of the computational power and modeling tools. Many of these models are based on relatively detailed single-cell models and data-driven connectivity structures, which allows to build simulations that can capture the specificities of local brain circuits (Markram, 2006; Hjorth et al., 2020; Gandolfi et al., 2022). Even when the advances have been remarkable, these detailed models demand high computational resources and are restricted to local circuits or brain regions, while building models at whole-brain level with single-cell resolution is still far from possible. Thus, an alternative solution that allows to move efficiently between scales (from cells to regions to whole-brain) is currently of great importance. One possibility has recently emerged which consists on using mean-field models of neuronal activity to build large-scale simulations (Sanz Leon et al., 2013; Sanz-Leon et al., 2015; Goldman et al., 2023). Mean-field models use statistical techniques to estimate the activity of large neuronal populations (from hundreds to thousands of neurons), which allows to reduce the dimensionality of the system. Thus, the activity of local brain circuits can be described in terms of a few differential equations, which drastically reduce the need of computational resources. The low-dimensionality of these models make them very good candidates to be integrated into large-scale simulations. Recently developed computational tools, such as the The Virtual Brain, make use of mean-field field models together with connectome information to build whole-brain simulations, and which can be performed without the need of large computational resources (Sanz Leon et al., 2013). This approach has been applied to whole-brain simulations for different species and is being used in basic research (Goldman et al., 2023; Stenroos et al., 2024) and for clinical applications (Bezgin et al., 2017; Hashemi et al., 2020), which shows the relevance and utility of these methods. Although the results obtained so far are notorious, these methods are normally based on generic mean-field models (sometimes inspired on cortical microcircuits), which do not incorporate the specificities of the different brain regions. However, the different activity patterns and functions that characterize each region is intrinsically linked to the specific cell-types and local connectivity structure observed in each area. Thus, in order to extend the utility and applicability of these methods it is of fundamental importance to incorporate the cellular heterogeneity and structural specificity observed in the brain. Some attempts in this direction have been done, mostly based on phenomenological mass-models adapted to capture particular dynamics (van Wijk et al., 2018; Levenstein et al., 2019), but which do not capture cell specificity and local connectivity structures. Only recently detailed mean-field models of a specific sub-cortical microcircuit have been proposed for the cerebellar cortex (Lorenzi et al., 2023), thalamus (Overwiening et al., 2023), and basal ganglia (Tesler et al., 2023a). Thus, further developments in this direction are of fundamental importance.

**Figure 1:**
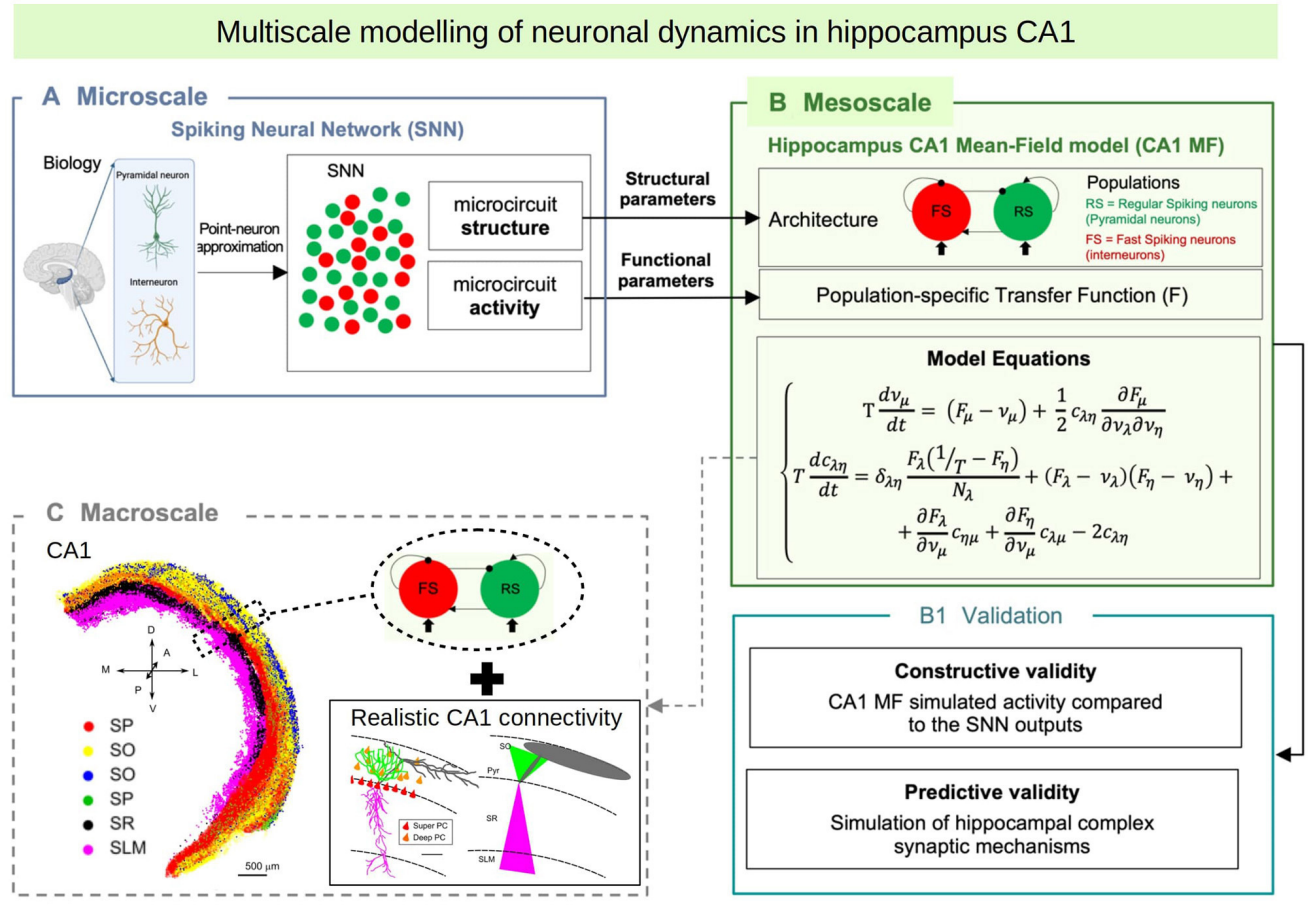
Diagram of the multiscale modeling framework. **(A)** Starting at the single-cell level, we build spiking neural networks taking into account cellular and local connectivity properties of hippocampus CA1. **(B)** We develop a mean-field model of the network dynamics using a recent bottom-up formalism, which incorporates the the cellular and network specificities. **(C)** Finally we build a macroscale model using the mean-field models (representing columns or local domains) in combination with realistic extended connectivity of CA1. The image of CA1 is adapted from Gandolfi et al. (2022). The color code represent different neurons in four layers of CA1: red: Superficial Pyramidal Cells (SP); yellow: Deep Pyramidal Cells (SO); blue: Stratum Oriens Inhibitory neurons (SO); green: Stratum Pyramidalis Inhibitory neurons (SP); black: Stratum Radiatum Inhibitory neurons (SR); magenta: Stratum Lacunosum Inhibitory neurons (SLM). For simplicity, in this paper we consider only Pyramidal cells and one type of inhibitory interneurons, but our framework can be extended to incorporate more cell types.

In this paper we introduce a multiscale modeling framework of the hippocampus which incorporates a newly developed mean-field model as the bridge between the different scales. In particular we focus on the hippocampal CA1, an area known for playing a key role in main brain functions such as learning, memory consolidation and navigation (O'Keefe and Nadel, 1978; Buzsáki, 1989; Moser et al., 2008). To develop the mean-field of the CA1 microcircuit we make use of a recently developed formalism that follows a bottom-up approach starting from the single-cell level, which allows to build a mean-field model that incorporates different cell types with specific intrinsic firing properties, and their synaptic interactions mediated by different receptor types (El Boustani and Destexhe, 2009; Zerlaut et al., 2018; Di Volo et al., 2019). In addition we develop a macroscale simulation of CA1 using the mean-field models as building blocks and incorporating extended specific connectivity structure based on a recently developed data-driven method (Gandolfi et al., 2022) (see Figure 1 for a diagram of the multiscale framework).

In the next sections we first present the model of the CA1 microcircuit and the mean-field formalism with more details and describe the development of the CA1 mean-field model. We test and validate our model by analyzing the multiscale model response under the main oscillatory activity observed in the hippocampus and comparing the mean-field model results with the ones of an equivalent spiking network model. Then, we analyze the implementation of synaptic plasticity within our framework, a key aspect to study the role of hippocampus in learning and memory consolidation. Finally we will show how the mean-field model can be used to build a macroscale simulation taking into account the realistic extended connectivity of CA1. The modeling framework presented here allows us to go from single-cell models to biologically realistic macroscale simulations while keeping a limited use of computational resources. In addition, our development is suitable to be incorporated into whole-brain simulation platforms (such as the TVB; Sanz Leon et al., 2013), which highlights the importance and usability of this approach.

## 2 Materials and methods

### 2.1 Single-cell model

Our multiscale modeling starts at the single-cell level. To perform single-cell simulations we adopt the Extended-Generalized Integrate-and-Fire neuronal model (EGLIF; Geminiani et al., 2018; Lorenzi et al., 2023). The equations for the EGLIF model describe the time evolution of membrane potential (*V*_*m*_), slow adaptation current (*I*_*adap*_) and fast depolarization current (*I*_*dep*_) (Equations 1–3):


(1) $$\frac{dV_{m}}{dt}=\frac{1}{C_{m}}(\frac{C_{m}}{\tau_{m}}(V_{m}(t)-E_{rev})-I_{adap}(t)+I_{dep}(t)+I_{e}+Isyn$$

(2) $$\begin{array}{r} \frac{dI_{adap}}{dt}=k_{adap}\left(V_{m}\left(t\right)-E_{rev}\right)-k_{2}I_{adap}\left(t\right) \end{array}$$

(3) $$\begin{array}{r} \frac{dI_{dep}}{dt}=k_{1}I_{dep}\left(t\right) \end{array}$$

where *I*_*syn*_ is the synaptic current modeling the synaptic stimulus, *C*_*m*_ is the membrane capacitance, τ_*m*_ is membrane time constant, *E*_*rev*_ is the reversal potential, *I*_*e*_ is the endogenous current, *k*_*adap*_ and *k*_2_ are adaptation constants and *k*_1_ is the decay rate of *I*_*dep*_. When a spike occurs at time *t*_*spk*_, the update rules of the state variables is given by Equations 4–6:


(4) $$\begin{array}{c} V_{m}\left(t_{\text{spk}}^{+}\right)=V_{r} \end{array}$$

(5) $$\begin{array}{c} I_{adap}\left(t_{\text{spk}}^{+}\right)=I_{adap}\left(t_{spk}\right)+A_{2} \end{array}$$

(6) $$\begin{array}{c} I_{dep}\left(t_{\text{spk}}^{+}\right)=A_{1} \end{array}$$

where $t_{spk}^{+}$ is the time instant immediately following *t*_*spk*_, *V*_*r*_ is the reset potential, and *A*_1_ and *A*_2_ are the model currents update constants. For our simulations we will consider only two types of cells [pyramidal cells and fast spiking interneurons (FS)], although the model could be extended to incorporate more cell types. Regarding the selection of our single cell-model, we note that a data-driven adaptive GLIF model (AGLIF) has been recently developed (Marasco et al., 2023), specifically conceived to capture the detailed dynamics observed experimentally in CA1 neurons and interneurons. In this work, we used a simplified EGLIF implementation, which is more easily adaptable to the multiscale formalism introduced in this paper while still provides an effective way of simulating the neuronal and population dynamics as will be shown in the next sections. The model parameters used for each cell type are given in Table 1. The mean-field formalism used for the analysis in the following sections has shown to be robust for large variations in neuronal parameters (Di Volo et al., 2019; Alexandersen et al., 2024), for which the specific cellular parameters used here serve as a general reference for building our system.

**Table 1:** Neuronal parameters for the EGLIF model.

	**Pyramidal cells (Pyr)**	**Interneurons (FS)**
*C*_*m*_ (pF)	2,877.83	2,939.66
τ_*m*_ (ms)	10,955.36	2,169.40
*E*_*rev*_ (mV)	-70.07	-74.01
*k*_*adap*_ (MH^−1^)	0.0084	0.0616
*k*_1_ (kHz)	0.0007	0.0021
*k*_2_ (kHz)	0.0042	0.0098
*A*_1_ (pA)	26.0	92.0
*A*_2_ (pA)	170.0	5.0
*I*_*e*_	0	0

We consider two cell types, pyramidal neurons (Pyr) and fast spiking inteneurons (FS).

Regarding the synaptic input, we consider a conductance-based interaction and we write Equation 7:


(7) $$\begin{array}{l} I_{syn}=G_{syn}^{e}\left(E_{e}-V_{m}\right)+G_{syn}^{i}\left(E_{i}-V_{m}\right), \end{array}$$

where *E*_*e*_ = 0mV (*E*_*i*_ = −80mV) is the excitatory (inhibitory) reversal potential and $G_{syn}^{e}$ ($G_{syn}^{i}$) the excitatory (inhibitory) synaptic conductance. When a presynaptic spike of neuron of type *j* occurs at time *t*_*spk*_, the conductance is modified according an alpha-function (Equation 8):


(8) $$\begin{array}{l} G_{syn}^{j}\left(t\right)=Q_{j}\frac{t-t_{spk}}{\tau_{syn}}\;e^{1-\frac{t-t_{spk}}{\tau_{syn}}\;}, \end{array}$$

where *Q*_*j*_ is the quantal conductance of type *j* (maximum conductance change per spike) and τ_*syn*_ is the synaptic characteristic time. We adopt *Q*_*Pyr*_ = 1.5 nS, *Q*_*FS*_ = 8.0 nS and τ_*syn*_ = 5 ms, respectively.

### 2.2 CA1 microcircuit and mean-field formalism

The second scale of our modeling framework is at the microcircuit level. For simplicity we will assume that the circuit is made of two cell-types, pyramidal excitatory cells (Pyr) and fast spiking inhibitory interneurons (FS), where each cell will be modeled with an E-GLIF model presented in the previous section. For the initial construction of the model we will consider a network of 5,000 Pyr-cells and 500 FS-cells (Aika et al., 1994; Bezaire and Soltesz, 2013; Ramirez-Villegas et al., 2018). Neurons in the circuit are interconnected with probability *p*_*Pyr*−*Pyr*_ = 0.01, *p*_*FS*−*Pyr*_ = 0.3, *p*_*Pyr*−*FS*_ = 0.2, *p*_*FS*−*FS*_ = 0.3 (Ramirez-Villegas et al., 2018; Tecuatl et al., 2021). The local microcircuit receives external excitatory input from the CA3 area, which will be modeled as an external poissonian input representing 5,000 excitatory neurons. The external input targets both Pyr and FS cells with probability of *p*_*ext*−*Pyr*_ = 0.15 and *p*_*ext*−*FS*_ = 0.3, respectively (Ramirez-Villegas et al., 2018).

Next, we introduce the mean-field model of the CA1 microcircuit dynamics. To develop this mean-field model we will adopt a recent formalism adapted for EGLIF neurons. The formalism is based on a bottom-up approach, starting at single-cell level, which allows the construction of mean-field models with cellular-type specificity. The second-order mean-field equations for the E-GLIF network are given by Lorenzi et al. (2023) (Equations 9, 10):


(9) $$\begin{array}{l} T\frac{d\nu_{\mu }}{dt}=\left(F_{\mu }-\nu_{\mu }\right)+\frac{1}{2}c_{\lambda \eta }\frac{\partial^{2}F_{\mu }}{\partial \nu_{\lambda }\partial \nu_{\eta }} \end{array}$$

(10) $$\begin{array}{l} T\frac{dc_{\lambda \eta }}{dt}=\delta_{\lambda \eta }\frac{F_{\lambda }\left(1/T-F_{\eta }\right)}{N_{\lambda }}+\left(F_{\lambda }-\nu_{\lambda }\right)\left(F_{\eta }-\nu_{\eta }\right)\\ \;+\frac{\partial F_{\lambda }}{\partial \nu_{\mu }}c_{\eta \mu }+\frac{\partial F_{\eta }}{\partial \nu_{\mu }}c_{\lambda \mu }-2c_{\lambda \eta }, \end{array}$$

where ν_*j*_ is the mean neuronal firing rate of the population *j*, *F* is the neuron transfer function (i.e., output firing rate of a neuron when receiving the corresponding excitatory and inhibitory inputs with mean rates $\nu_{j}^{\prime }s$), and *T* is a characteristic time for neuronal response (we adopt *T* = 5 ms). In this equation μ, ν, λ = {*Pyr, FS*} and the Einstein index notation was used, where repeated indices imply a summation over all the values of the index. Finally, *c*_λ, ν_ represents the covariance between the activity of neuronal populations λ, ν. The value used for the characteristic time *T* is linked to the autocorrelation time of the system (see El Boustani and Destexhe, 2009 for details).

Following Zerlaut et al. (2018) we write the transfer function for each neuronal type as Equation 11:


(11) $$\begin{array}{l} F_{\nu }=\frac{1}{2\tau_{V}}erfc\left(\frac{V_{\text{thre}}^{\text{eff}}-\mu_{V}}{\sqrt{2}\sigma_{V}}\right), \end{array}$$

where *erfc* is the error function, $V_{\text{thre}}^{\text{eff}}$ is an effective neuronal threshold, μ_*V*_, σ_*V*_ and τ_*V*_ are the mean, standard deviation and correlation decay time of the neuronal membrane potential. The effective threshold can be written as a second order polynomial expansion (Equation 12):


(12) $$V_{\text{thre}}^{\text{eff}}(\mu_{V},\sigma_{V},\tau_{V}^{N})=P_{0}+\sum_{x\in \{\mu_{V},\sigma_{V},\tau_{V}^{N}\}}P_{x}\cdot (\frac{x-x^{0}}{\delta x^{0}})+P_{\mu_{G}}\ln(\frac{\mu_{G}}{g_{L}})$$

where *x*^0^, δ*x*^0^ are constants, the coefficients *P*_*x*_ are to be determined by a fit over the numerical transfer function obtained from single-cell spiking simulations for each specific cell-type, and where μ_*G*_ is given by Equation 13:


(13) $$\begin{array}{l} \mu_{G}=\underset{j}{\sum }\left(Q_{j}\tau_{j}\nu_{j}K_{j}\right)+g_{L} \end{array}$$

with *K*_*j*_ = *p*_*i*−*j*_*N*_*j*_ the mean synaptic convergence of type *j*, being *N*_*j*_ the number of cells of this type.

We can write the mean membrane potential and standard deviation as (Lorenzi et al., 2023) (Equation 14):


(14) $$\begin{array}{l} \mu_{V}=e\frac{\underset{j}{\sum }\mu_{Gj}E_{j}+g_{L}E_{L}}{\mu_{G}} \end{array}$$

Finally, the standard deviation and correlation decay time of the neuronal membrane potential can be written as Equations 15, 16:


(15) $$\begin{array}{l} \sigma_{V}=\sqrt{\underset{j}{\sum }K_{j}\nu_{j}\left(2\tau_{m}^{eff}+\tau_{j}\right){\left(\frac{eU_{j}\tau_{j}}{2\left(\tau_{m}^{eff}+\tau_{j}\right)}\right)}^{2}} \end{array}$$

(16) $$\begin{array}{l} \tau_{V}=\frac{\underset{j}{\sum }K_{j}\nu_{j}{\left(eU_{j}\tau_{j}\right)}^{2}}{2\underset{j}{\sum }K_{j}\nu_{j}\left(2\tau_{m}^{eff}+\tau_{j}\right){\left(\frac{eU_{j}\tau_{j}}{2\left(\tau_{m}^{eff}+\tau_{j}\right)}\right)}^{2}} \end{array}$$

with $\tau_{m}^{eff}=\frac{C_{m}}{\underset{j}{\sum }\mu_{Gj}+g_{L}}$ and $U_{j}=\frac{Q_{j}}{\underset{j}{\sum }\mu_{Gj}+g_{L}}\left(E_{j}-\mu_{V}\right)$, where *Q*_*j*_ is the quantal conductance of type *j* and *C*_*m*_ is the membrane capacitance.

The details of the derivation of the mean field equations can be found in Di Volo et al. (2019) and Lorenzi et al. (2023).

## 3 Results

We start the construction and validation of our multiscale modeling framework with the estimation of the transfer function parameters, needed for the implementation of our mean-field model of CA1. Then we validate this model by comparison with spiking network simulations, for different situations, and we terminate by showing the simulation of mesoscale phenomena such as traveling waves in large-scale systems.

### 3.1 Mean-field model of CA1 microcircuit

**Figure 2:**
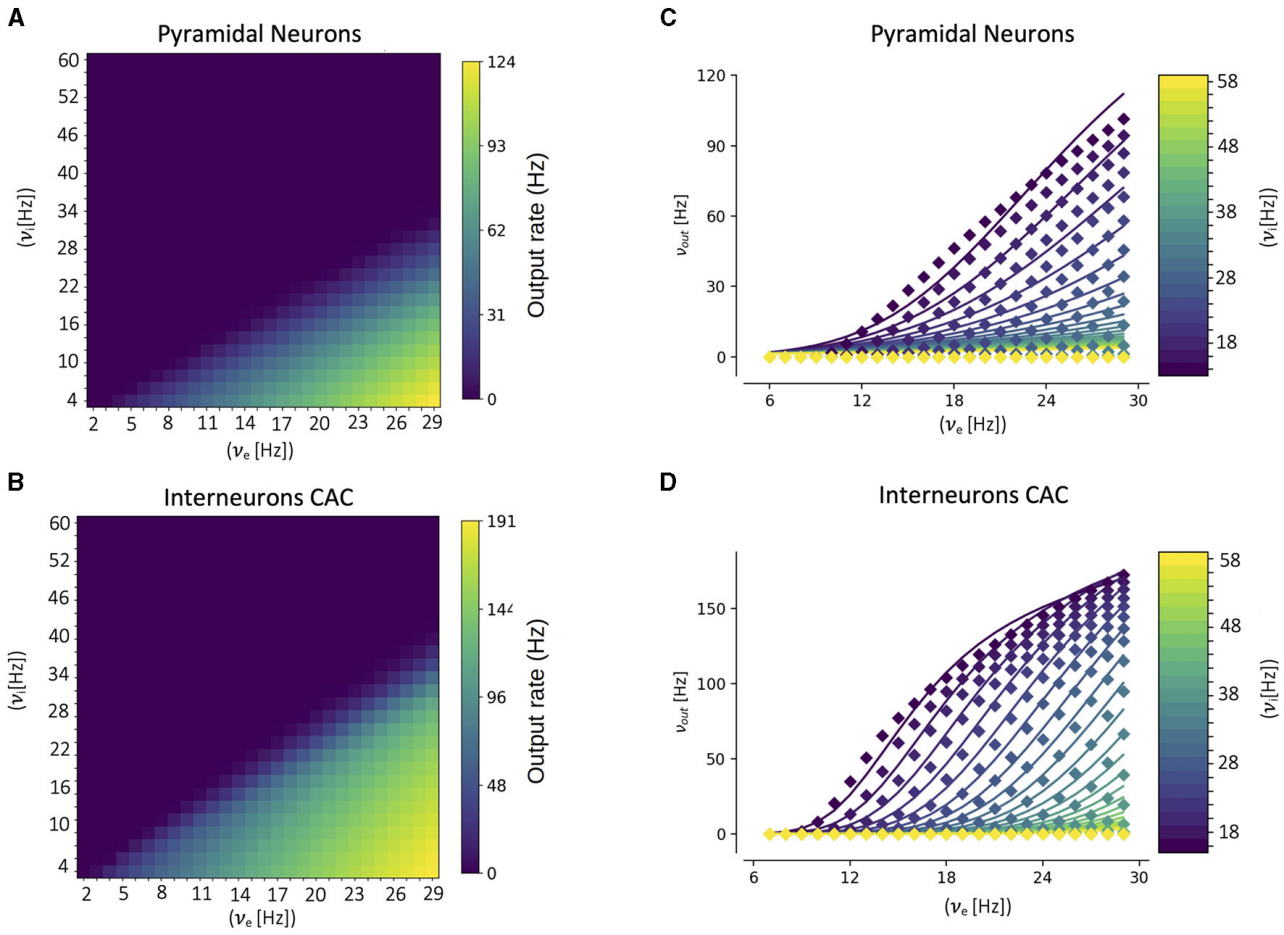
Numerical transfer function **(A, B)** and the corresponding semi-analytical approximation fitted from Equation 11 **(C, D)** for each cell-type. Solid lines in **(C, D)** correspond to the firing rates obtained from Equation 11 while filled-squares correspond to the single-cell numerical results. The input firing rates (ν_*e*_, ν_*i*_) in the single-cell case correspond to the mean rates of a poissonian process simulating the excitatory and inhibitory neuronal inputs, respectively.

A key component of our multiscale framework is the incorporation of the mean-field model of the CA1 dynamics. As explained in the previous section, in the center of the mean-field formalism is the utilization of a semi-analytical transfer function. Thus, to build the mean-field model for CA1 the first step is to calculate the corresponding transfer function for each cell type. This is done by fitting the numerical transfer function obtained from single-cell simulations to the semi-analytical expression of the transfer function (Equation 12). In Figure 2 we show the results of the numerical transfer function together with the corresponding fit for each cell-type (pyramidal cells and interneurons). We can see that our semi-analytical transfer function can correctly captured the one obtained numerically. Once the parameters of the semi-analytical transfer functions are obtained, together with the cellular and network parameters (as described in the presentation of the formalism in the previous section), the mean-field model is fully defined.

### 3.2 Activity patterns and time varying inputs

**Figure 3:**
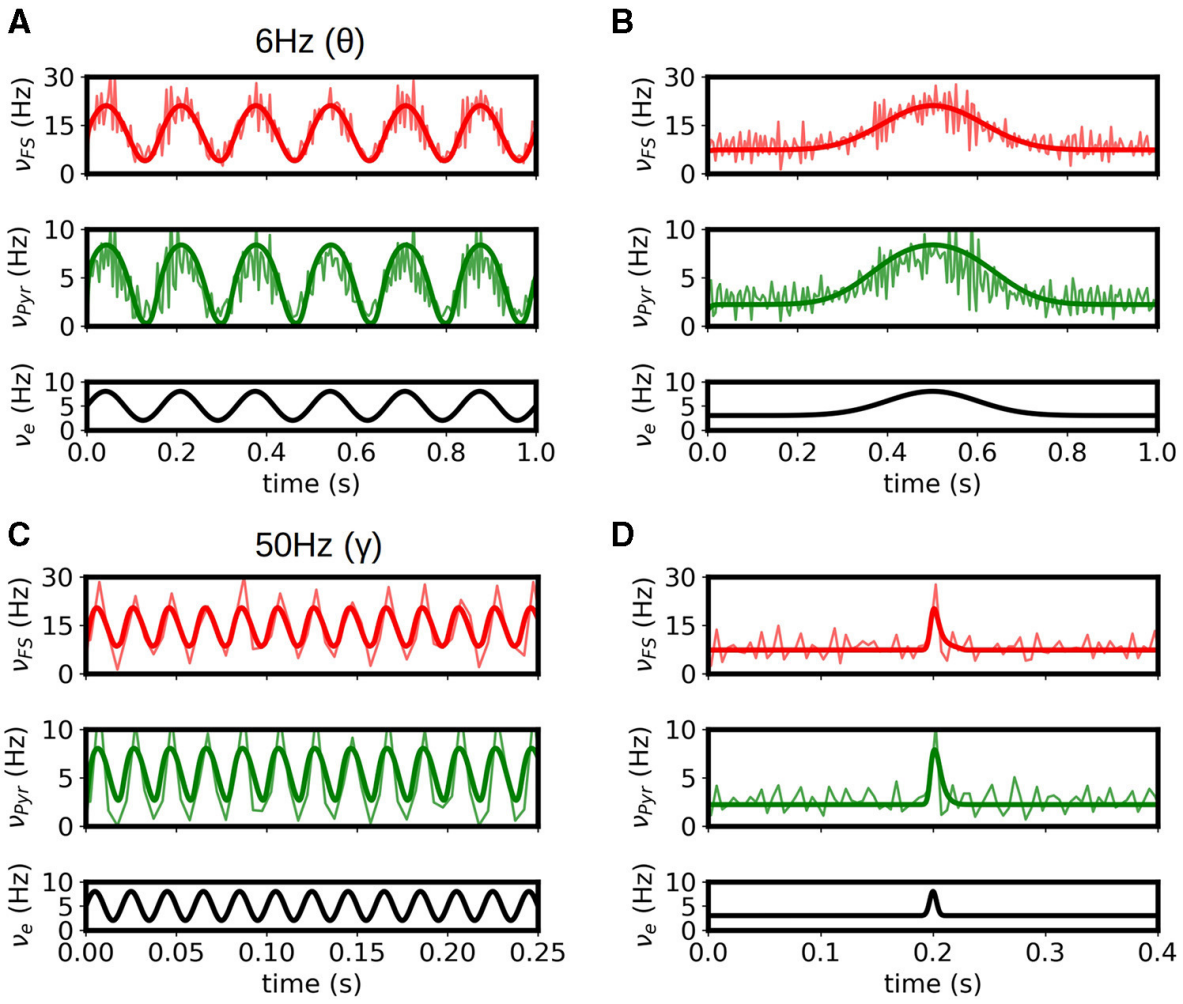
Response of the systems to θ **(A)** and γ **(B)** rhythms. Results from the mean-field (bold solid lines) are superimposed to the firing rates obtained from the spiking-network (SNN) simulations of the hippocampus (light solid lines). **(C, D)** Response of the system to slow and fast Gaussian inputs. We see that the mean-field can capture the response of the SNN in large frequency-range (from 6 Hz in θ waves to ~140 Hz for the fast Gaussian input), relevant to simulate the different activity patterns observed in the hippocampus. For high frequency the accuracy of mean-field is reduced as the typical time of variation in activity gets closer to the characteristic times of the mean-field.

To validate the multiscale model of the hippocampus we test the response of the model to some of the main activity patterns observed in CA1. It is well established that three main patterns of activity are present in the hippocampus and can be observed during specific brain states: theta oscillations (4-10Hz) are normally associated with exploratory behavior, sharp-wave/ripple complexes (140–200 Hz) are associated with immobility, and gamma oscillations (40-140 Hz) are normally present in combination and modulated by the other two rhythms. In Figures 3A, B we show results of the simulation for stimulations on the theta and gamma ranges. We show the results obtained with mean-field superimposed to the results from the spiking neural network (SNN). As we can see the mean-field can correctly reproduce the response of the system for the different input patterns.

In addition, in Figures 3C, D we show the response of the system to low and fast Gaussian-shaped inputs. The fast input can be seen as similar to the activity of sharp-waves in CA1, while the slow input can be seen as a typical response curve of place cells in CA1 for space-field selectivity. The mean-field is capable of capturing the response of the system for both cases. For fast or high-frequency inputs the accuracy of mean-field is slightly reduced as the typical time of variation in activity gets closer to the characteristic times of the mean-field.

### 3.3 Synaptic plasticity

**Figure 4:**
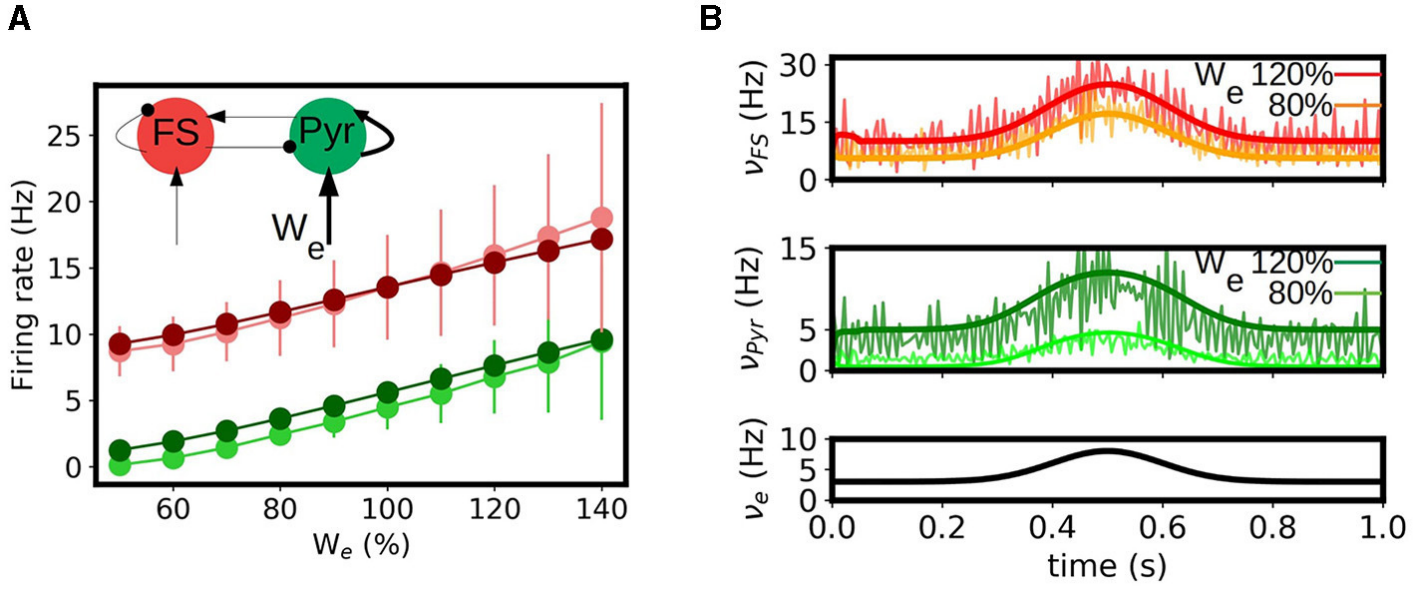
Synaptic potentiation and depression in the mean-field model. **(A)** Evolution of the response of pyramidal and fast spiking cells as a function of the strength of synaptic convergence (*W*_*e*_). We show the results obtained for the mean-field model (dark green and dark red respectively circles) and the spiking neural network (light green and light red) for a constant external input of ν_*e*_ = 5Hz. A level of *W*_*e*_ = 100% correspond to the baseline level (described in the Section 2). Inset: diagram of the network and indication of the change in convergence. **(B)** Time varying inputs for two levels of *W*_*e*_. We show the firing rates of the FS and Pyr cells obtained from the mean-field and the spiking network together with the applied input (ν_*e*_).

The occurrence of long term synaptic depression (LTD) and potentiation (LTP) in the hippocampus was among the first experimental studies presented on long term synaptic plasticity and is believed to be related with the role of hippocampus in learning and memory formation, one of the main known functions of this region (Bear and Malenka, 1994; Malenka and Bear, 2004). Thus, the capacity of reproducing the effects of synaptic changes in neuronal activity is a key feature to be captured by a model of this region. To perform this study we analyze the response of our mean-field model under variations in the synaptic convergence (*K*, see Section 2). In particular we consider variations in the synaptic convergence of the simulated CA3 afferent input to the local Pyramidal cells in CA1 (see diagram in Figure 4A). We introduce the parameter *W*_*e*_ which quantifies the changes in the weight of the synaptic convergence, being *W*_*e*_ = 100% the baseline level (as considered in the previous sections), and we analyze the response of the system for a variation in the range of 50% in the strength of the synaptic convergence for a constant input and a time varying input. In Figure 4A we show the evolution of the response of pyramidal cells as a function of *W*_*e*_ and its comparison with the results from the spiking neural network. We can see that, although there is a small overestimation of the activity for certain values of *W*_*e*_, the mean-field model can correctly capture the evolution of the response obtained in the spiking network. In Figure 4B we show the response of the mean-field and spiking network under a time-varying input of Gaussian shape for two different levels of *W*_*e*_ (*W*_*e*_ = 80 and 120%). As we can see the mean-field can correctly reproduce the response of the network for the different values of *W*_*e*_. We note how the response of the neuronal populations to the changes in *W*_*e*_ is different for each cell-type, which becomes more evident for the lower values of *W*_*e*_. This is a direct consequence of the non-linear response characterizing each neuronal type, which is in particular captured by each corresponding transfer function. This aspect further shows the importance of incorporating the cellular specificities within the mesoscale description for accurately modeling different phenomena, as done within our approach.

Finally, we note that, as a first approach, we only considered variations in the synaptic convergence, which allowed us to analyze in a general way the impact of the change of synaptic properties in the neuronal activity in our model. However, further analysis can be done around other synaptic parameters with our approach, such as the quantal conductance (*Q*_*j*_) or the synaptic decay times, and the modeling of specific receptors as it has been recently shown (Lorenzi et al., 2023; Tesler et al., 2023b).

### 3.4 Detailed connectivity structure and macro-scale simulations of the CA1 network

**Figure 5:**
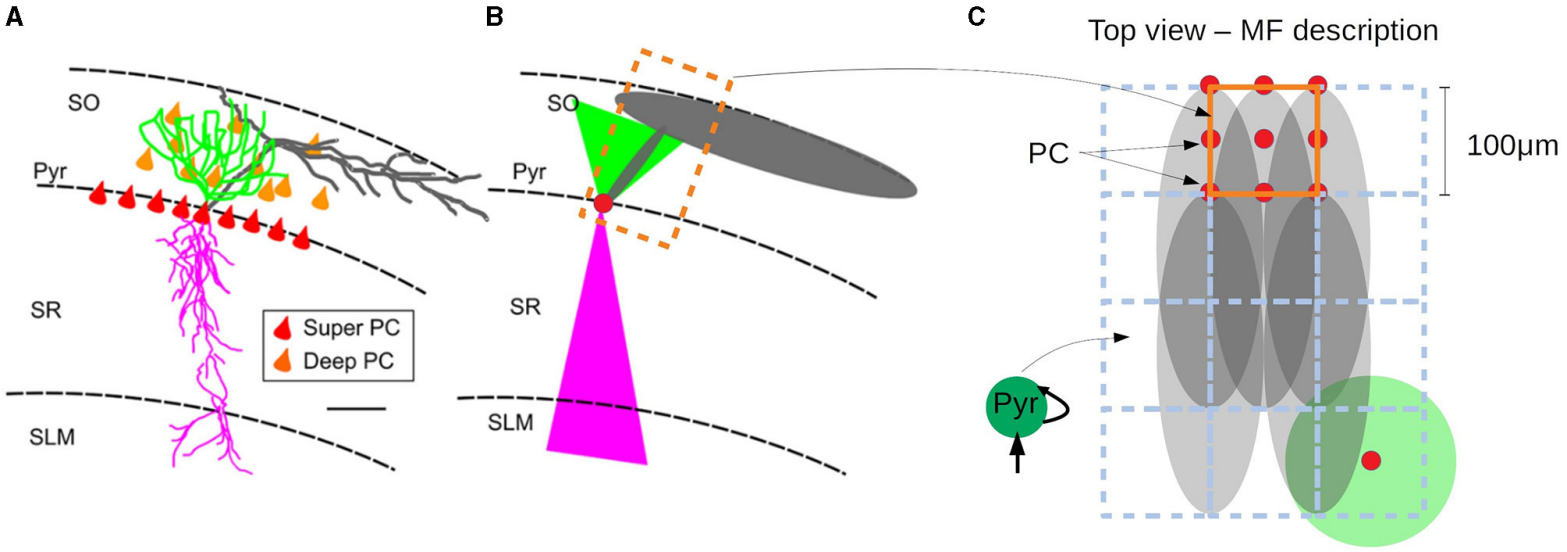
**(A)** Realistic morphology of a superficial pyramidal cell (PC) with basal dendrites in green, apical in pink, and axon in gray, oriented within a region of a transversal CA1 hippocampal slice. Red triangles correspond to PC soma location within the stratum pyramidalis whereas orange triangles represent the scattered distribution of deep PCs within the SO. **(B)** Probability clouds of connectivity represented as two triangles (2D of a cone) and an ellipse (2D of an ellipsoid). Color code respects the realistic morphology. The dashed rectangle in dark-orange corresponds to the area covered by a single mean-field compartment described in **(C)**. **(C)** Top-view of **(A, B)** and diagram of the compartmentalization for the mean-field description of the hippocampal network with the corresponding single-cell probability clouds. Color code follows **(A, B)**. Axonal probability clouds are shown for five pyramidal cells (with somas indicated in red-circles) located at the border of a compartment (indicated in dark-orange). Neighboring compartments are shown in dashed blue lines. Probability cloud for basal dendrites of single PC cell is shown at the bottom right with the soma located at the center of the compartment (red circle). **(A, B)** are adapted from Gandolfi et al. (2022).

In this section we show an example of the passage from the mesoscale to the macroscale with the use of the mean-field model. As discussed before, one of the main goals of our approach is to build a model of a specific area with realistic connectivities based on available physiological, morphological and anatomical data. In this section we will present the results of simulations of a network representing a slice of hippocampal CA1 area. To this end we will adopt a recently developed method to incorporate realistic morpho-anatomical connectivities based on the geometrical probability volumes associated with pre- and postsynaptic neurites (Gandolfi et al., 2022). The method has been benchmarked for the mouse hippocampus CA1 area, and the results show that this approach is able to generate full-scale brain networks that are in good agreement with experimental findings. Following Gandolfi et al. (2022), we will focus on a particular case where only excitatory connections are taken into account, a case which has been previously compared to experimental results. In Figures 5A, B we show a diagram of the geometric probability volume associated with pyramidal cells and the distribution of Pyr cells in CA1, adapted from Gandolfi et al. (2022). We will assume that the Pyr cells are homogeneously distributed over the Pyr and SO layers. The geometric probability volumes associated with the basal, apical dendrites and axon are indicated in green, pink, and gray, respectively. Axonal volumes can be represented by a combination of two elliptical volumes, while dendritic volumes can be represented by conical volumes. The most relevant region for Pyr-to-Pyr connectivity lies within the Pyr-SO region, we will therefore concentrate our attention on this area to build our network. We will consider a slice covering a surface of 1.5 x 1.5 mm^2^ along the Pyr-SO layer. We will divide this area in compartments of 100μ*m*x100μ*m* containing about 200 neurons each and we will describe each of this compartment with a single mean-model as described in the previous sections. To build the connectivity between compartments we will make use of the geometric probability volumes. In Figure 5C we show a diagram of the compartmentalization and the corresponding single-cell probability volumes. The connectivity between compartments (given by the parameter *K* in Equation 13) will be defined as proportional to the normalized probability of connections given by the probability volumes. Here we assume that the dendritic volumes extend through the entire transverse length of the Pyr-SO layer, for which we assume that the main constraint for the connectivity is given by the axonal volume (see Figure 5).

**Figure 6:**
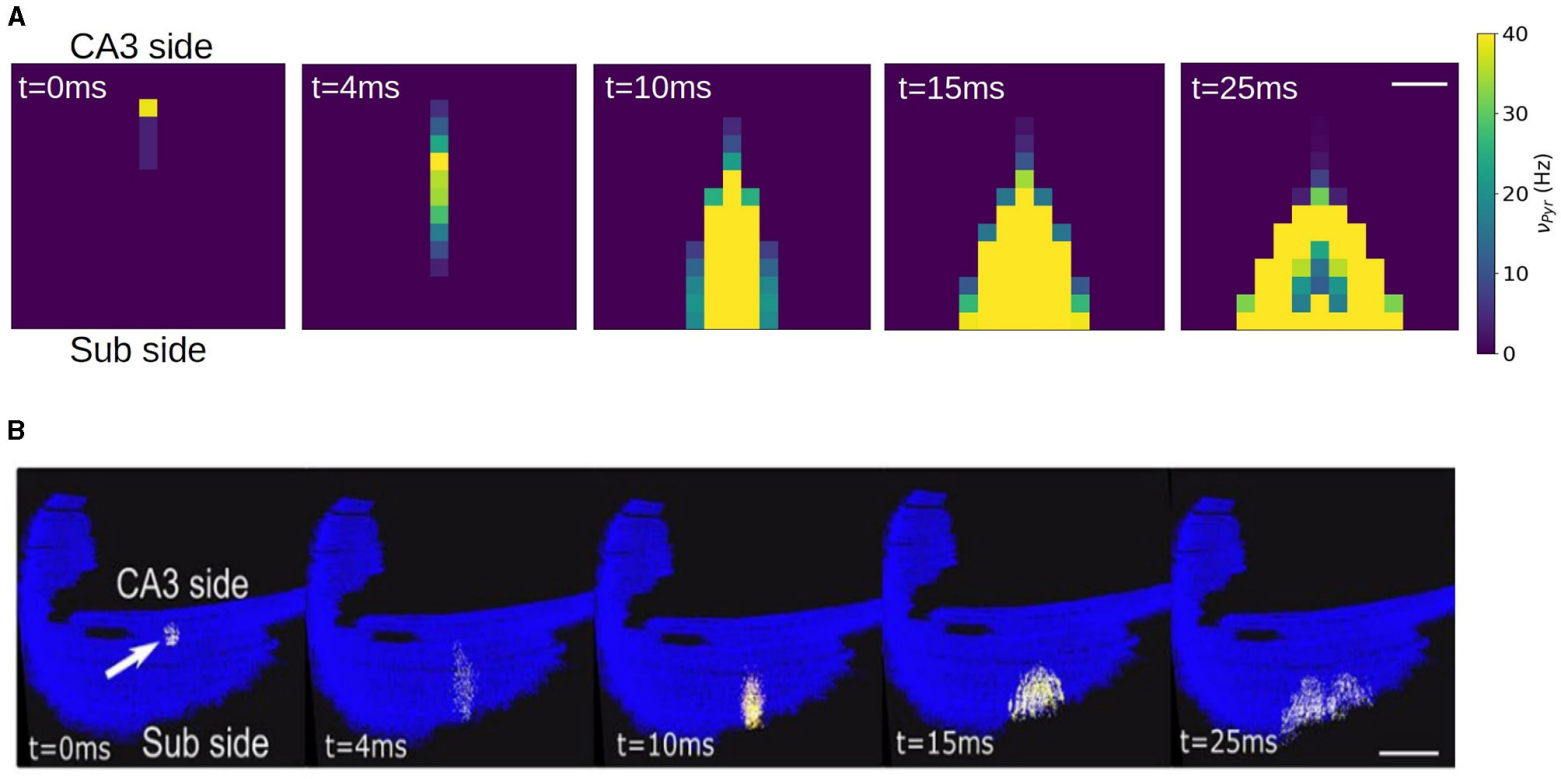
Simulation of a local stimulation in a CA1 network. Activity is evoked near the CA3 side in area of 1e4 μ^2^ containing ~200 pyramidal neurons, represented by a single mean-field model. The stimulation induces a rapid propagation of the activity in the transversal direction (antero-posterior) of the network (4.10 ms) with a gradual increase in neuronal recruitment and a subsequent propagation in the longitudinal direction (medio-lateral). The network correspond to a slice of 1.5 x 1.5 mm. Firing rates are indicated on the colorbar. Scale bar 300 μ*m*. **(B)** Stimulation protocol equivalent to **(A)** performed in a full CA1 spiking network, adapted from Gandolfi et al. (2022). Scale bar 1 mm. Activity is color coded from blue (rest) to white (spike), to visualize action potentials, with a fixed 2 ms transition time.

It has been shown experimentally that in the absence of synaptic inhibition CA1 activity shows strong directionality from the CA3 side to the subiculum side. This has been also reproduced by spiking network simulations of CA1 following the same geometric connectivity volume approach. To validate our network we show in Figure 6 the results from the mean-field network slice together with the results from the corresponding spiking network simulation. In this simulations a short stimulus is applied to a single compartment in the case of the mean-field and to ~ 200 neurons close to the CA3 region. As we can see the connectivity profile induces a strongly directed propagation from the CA3 to the Subiculum direction. In addition, the propagation evolves with an increase in neuronal recruitment which in turns leads to the appearance of a lateral propagation as the activity gets closer to the CA1-Subiculum edge. These two features can be well captured by the mean-field network.

## 4 Discussion

In this paper we have introduced a multiscale modeling framework of the CA1 microcircuit, which goes from the single-cell to the macroscale level. This framework incorporates a newly developed mean-field model that allow us to perform an efficient passage between the different scales. The mean-field model was built using a recently introduced formalism that follows a bottom-up approach, starting at the single-cell level, which made possible to incorporate cellular and synaptic specificities of CA1 within the mean-field formulation. The single-cell parameters were based on previous detailed data-based modeling of CA1 pyramidal neurons and fast-spiking interneurons (Marasco et al., 2023), and synaptic connectivity information was based on experimental data (Ramirez-Villegas et al., 2018; Tecuatl et al., 2021). We have tested the model by analyzing its response under different oscillatory rhythms found in CA1 and we have validated the results by comparison with the corresponding spiking network model. We have shown in Section 3.2 that the mean-field is capable of capturing the results of the spiking-network for activity patterns related with some of the main patterns observed in CA1 (theta oscillations, sharp-waves and gamma oscillations).

In addition, we have explored how variations at the synaptic level can be captured by our model, which is a key aspect to incorporate in a model of this region. Although this represents a simple illustration of the use of our model for studying synaptic changes, we note that the analysis can be extended to other synaptic parameters within our approach, such as the quantal conductance (*Q*_*j*_) or the synaptic decay times, and the modeling of specific receptors as it has been recently shown (Lorenzi et al., 2023; Tesler et al., 2023b).

Finally we have shown an example of the implementation of a macroscale simulation within our framework. In particular we built a simulation of a slice of CA1 with specific connectivity structure, based on a recently developed data-driven method (Gandolfi et al., 2022). Furthermore, we compared the results of our simulations with an equivalent simulations of a spiking-network model of CA1, showing that our model can capture some of the main features of the spiking simulations, which further validates our model.

Among the limitations of our approach we notice that the connectivity between local populations is assumed to be random within the mean-field formalism. A possible solution to build systems with specific connectivity structures consists in the combination of multiple mean-field models (with random local connectivity, but structured longer range connectivity), as done in Section 3.4 of our paper. In addition, the introduction of heterogeneity within this mean-field formalism has been studied in a recent paper (Di Volo and Destexhe, 2021).

The modeling framework presented in this work is a step forward to the development of region-specific multiscale models. In addition, the framework developed here is suitable to be included in whole-brain simulation platforms (Sanz Leon et al., 2013), which extends the importance and utility of our study. Furthermore, methods to estimate brain signals (LFP, EEG, MEG, and fMRI) from the type of mean-field used here have already been developed (Tesler et al., 2022, 2023b), which will also allow the comparison with experimental results on whole-brain activity. In combination, these developments provide an efficient solution to the complicated task of modeling the brain at different scales and open new perspectives for future studies.

## Data availability statement

The raw data supporting the conclusions of this article will be made available by the authors, without undue reservation. The computational code used to perform the simulations presented in the paper is available at: https://doi.org/10.5281/zenodo.12653912.

## Author contributions

FT: Conceptualization, Formal analysis, Investigation, Methodology, Software, Writing – original draft, Writing – review & editing. RL: Conceptualization, Formal analysis, Investigation, Methodology, Software, Writing – original draft, Writing – review & editing. AP: Conceptualization, Investigation, Methodology, Writing – review & editing. CC: Conceptualization, Investigation, Methodology, Supervision, Writing – review & editing. FP: Investigation, Methodology, Software, Writing – review & editing. DG: Investigation, Methodology, Writing – review & editing. CG: Conceptualization, Investigation, Methodology, Supervision, Writing – review & editing. JM: Investigation, Methodology, Writing – review & editing. ED'A: Conceptualization, Funding acquisition, Investigation, Methodology, Supervision, Writing – review & editing. MM: Conceptualization, Funding acquisition, Investigation, Methodology, Supervision, Writing – review & editing. AD: Conceptualization, Funding acquisition, Investigation, Methodology, Supervision, Writing – original draft, Writing – review & editing.
